# When rebuilding no longer means recovery: the stress of staying put after Hurricane Sandy

**DOI:** 10.1007/s10584-021-03069-1

**Published:** 2021-04-23

**Authors:** Liz Koslov, Alexis Merdjanoff, Elana Sulakshana, Eric Klinenberg

**Affiliations:** 1 Department of Urban Planning and Institute of the Environment and Sustainability, University of California, Los Angeles, Los Angeles, CA 90095 USA; 2 School of Global Public Health, New York University, New York, 10003 NY USA; 3 Undergraduate Sustainable Development Program, Columbia University, New York, 10027 NY USA; 4 Department of Sociology, New York University, New York, 10003 NY USA

**Keywords:** Climate change, Disaster, Housing, Mental health, Relocation

## Abstract

After a disaster, it is common to equate repopulation and rebuilding with recovery. Numerous studies link post-disaster relocation to adverse social, economic, and health outcomes. However, there is a need to reconsider these relationships in light of accelerating climate change and associated social and policy shifts in the USA, including the rising cost of flood insurance, the challenge of obtaining aid to rebuild, and growing interest in “managed retreat” from places at greatest risk. This article presents data from a survey of individuals who opted either to rebuild in place or relocate with the help of a voluntary home buyout after Hurricane Sandy. Findings show those who lived in buyout-eligible areas and relocated were significantly less likely to report worsened stress than those who rebuilt in place. This suggests access to a government-supported voluntary relocation option may, under certain circumstances, lessen the negative mental health consequences associated with disaster-related housing damage.

## Introduction

Hurricane Sandy was one of many recent disasters to prompt an emerging conversation on “managed retreat,” the planned relocation of populations from high-risk areas exposed to accelerating effects of climate change (Hino et al. 2017; Koslov 2016; Siders et al. 2019). Sandy’s track up the densely populated east coast of the USA damaged or destroyed approximately 357,000 housing units along the New York and New Jersey coastlines, exceeding $50 billion dollars in damage (Sullivan and Uccellini 2013; NJ 2013; NY 2013). Contributing to these costs was 1 ft of sea-level rise over the decades before Sandy struck, estimated to have added approximately $2 billion to the storm’s toll in New York City and to have flooded an extra 100,000 people in New York and New Jersey (Horton et al. 2015).

Escalating risks posed by unmitigated climate change, plus prior experience of flooding, provoked much discussion after Sandy of whether and how to rebuild. Nationally, one survey found that 65% of Americans favored government-funded rebuilding while a smaller majority (53% nationwide; 59% in Sandy-affected areas) favored government-funded relocation and buyout policies (AP-NORC 2013). Among Sandy-affected neighborhoods, attitudes toward relocation and buyouts were mixed. Some households and groups of neighbors expressed a desire to move; others vocalized commitment to staying (Binder et al. 2015; Solecki et al. 2017). Government responses were likewise divided. New York and New Jersey launched home buyout programs in select areas while localities like New York City pursued rebuilding-focused recovery with no buyout option.

It remains uncertain how decisions to pursue relocation versus rebuilding shape disaster recovery experiences of affected populations. Buyout programs, which support hazard-prone properties’ demolition and conversion to open space, are relatively novel. Though the USA has seen a number of buyouts (FEMA, the Federal Emergency Management Agency, funded more than 40,000 from 1989 to 2017), scholars are just beginning to examine their outcomes and implications, spurred by interest in their use as a tool to adapt to climate change (Mach et al. 2019; Elliott et al. 2020). The bulk of research on post-disaster migration examines cases of sudden displacement and forced relocation, experiences of which may differ markedly from the managed, voluntary movement that buyout programs, in theory, enable. There are few direct comparisons between individuals who receive support to relocate and those who do not and scarce research into the experiences of such individuals and their communities over time (for important exceptions, see Binder et al. 2019; Barile et al. 2020). These gaps are crucial to address given disaster recovery’s nonlinear nature, and as climate change alters baseline conditions, making extreme events less exceptional and subjecting growing numbers to recurring trauma and dislocation (Arcaya et al. 2020; Klinenberg et al. 2020).

**Fig. 1:**
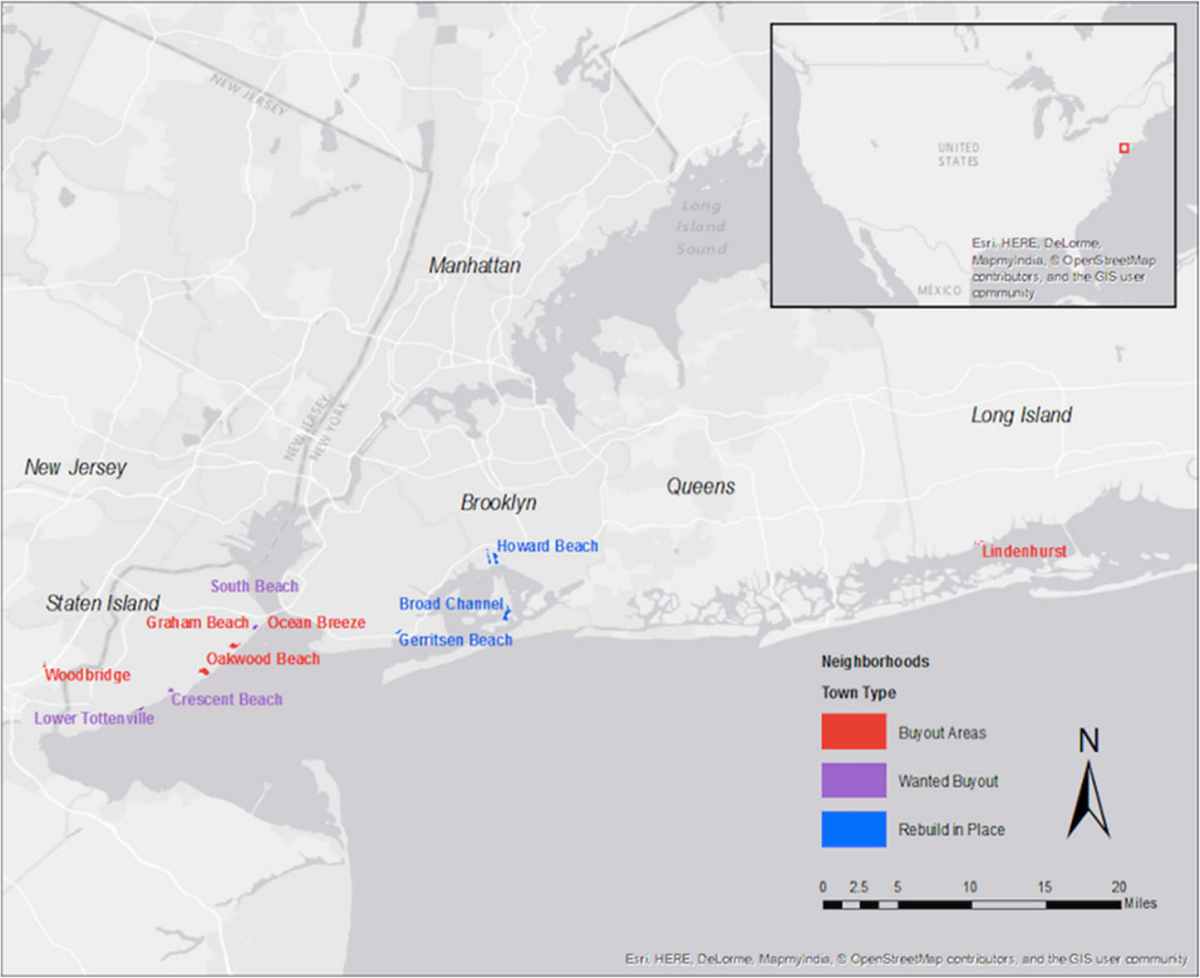
Map of survey sample areas constructed on GIS from Google Earth images provided by the Eagleton Center for Public Interest Polling. Buyout areas appear in red; wanted buyout areas, on Staten Island, in purple; rebuild in place areas, in Brooklyn and Queens, in blue

**Fig. 2:**
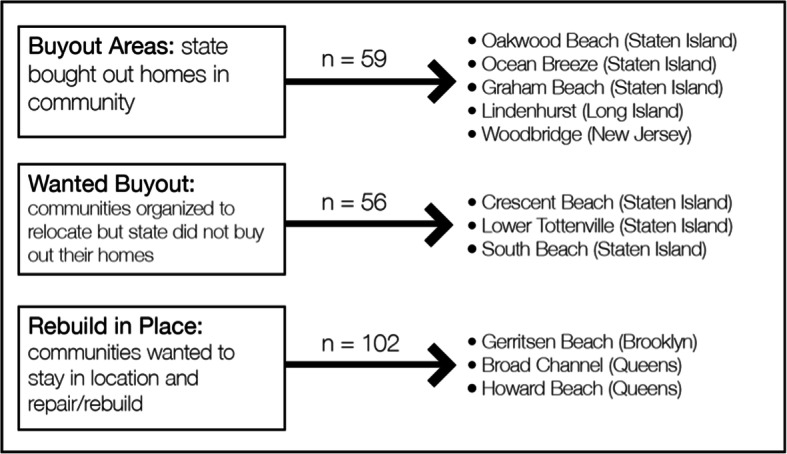
The 11 neighborhoods can be organized by the action residents collectively advocated post-Sandy, rather than what individuals opted to do. This takes into account community preferences, specifically buyout petitions that failed

To aid understanding of relocation versus rebuilding experiences over time in the context of climate change, this article draws on data from a targeted comparison of homeowners in three types of disaster-affected neighborhoods with differing approaches to housing recovery: *buyout areas*, selected after Sandy for government-funded buyouts based largely on homeowner demand; *wanted-buyout areas*, where some residents organized and lobbied for buyouts without success; and *rebuild-in-place areas* with no organized buyout effort and a public focus on rebuilding.^1^ These three types (see Fig. 1 for a map of sample areas and Fig. 2 for a table of areas in each category) shared similar demographic characteristics, Sandy damage, and future flood risk. Nonetheless, their residents expressed differing preferences for rebuilding versus retreat and received different housing recovery options from the government, allowing a comparison of experiences associated with these pathways.

The following section situates our study in the context of work that seeks to identify and explain the uneven outcomes for participants in post-disaster buyout and acquisition programs, relative to research on forced relocation as well as staying put amidst environmental change. We next describe our research design and methods, introducing the *Life on the Coast: Before and After Hurricane Sandy Study*, a cross-sectional survey we conducted three-and-a-half years after the storm. The study examined items related to respondents’ living situations before and after Sandy, including perceptions of their pre- and post-storm neighborhoods and their social, financial, and health circumstances. Findings revealed significant individual- and neighborhood-level differences that emerged during rebuilding and relocation, particularly in regard to self-reported stress. We conclude by discussing what these findings suggest about how experiences and outcomes of disaster recovery are shifting in light of more frequent and severe weather events, related policy responses, and anticipation of the worsening climate crisis.

## Assessing outcomes of retreat versus rebuilding

Despite growing interest in managed retreat in the USA (e.g., Flavelle 2020), the impacts of expanding and participating in relocation programs such as home buyouts—the primary means at present to implement retreat in the USA—remain unclear. Simultaneously, while rebuilding after a disaster has long been the favored scenario, prioritized and funded accordingly, the reality of climate change renders remaining in place newly problematic. What does it mean to stay or go in this context? How does managed retreat affect participants? How does it affect those who opt not to participate or whose movement is not facilitated in this way?

Substantial research links relocation to adverse social, economic, and physical and mental health outcomes but largely relies on cases of forced relocation and unplanned displacement following disasters (Riad and Norris 1996; Uscher-Pines 2009). Buyouts, by contrast, are legally voluntary, though may not be experienced that way (De Vries and Fraser 2012). Buyouts also often occur in response to homeowner demands, as after Sandy. Yet even in cases like this decisions to take a buyout are complex, involving various interacting individual, household-level, social, and contextual factors (Robinson et al. 2018; Seebauer and Winkler 2020). Decisions can change over time (Solecki et al. 2017), and participants may reevaluate their initial preference or look back with regret as they move through the process (Binder and Greer 2016; Baker et al. 2018). Understanding buyout outcomes requires attention to such longer-term trajectories and transformations, including the places where people move and those they leave behind. Many groups affected by retreat—renters, residents of mobile home parks (Rumbach et al. 2020), and people living in buyout-adjacent areas (Binder et al. 2020), among others—do not possess the same decision-making power or voice in the process as property owners offered buyouts. Like rebuilding, which can drive up housing prices and result in less visible waves of displacement (Pais and Elliott 2008), effects of retreat accrue at multiple temporal and spatial scales. Assessments of their outcomes depend on the vantage point and context.

Regarding retreat’s direct effects on participating households, findings from the nascent research on buyout outcomes are mixed, raising questions about how much retreat reduces risk versus displaces and redistributes it. One study examining the destinations of more than 300 Staten Island residents who took buyouts after Sandy estimated that a significant fraction, 21%, moved to locations at least as exposed to coastal flooding. The study also found that nearly all moved to census tracts measuring higher on a set of social vulnerability indicators. For example, 95%, relocated to areas with increased poverty rates (McGhee et al. 2020). Conversely, a study of more than 3000 buyouts in Harris County, Texas, found “a clear pattern of neighborhood upgrading,” with participants relocating to destination tracts with higher average incomes, median housing values, shares of owner-occupied housing, and nonpoor residents (Loughran and Elliott 2019, p. 65). This, the authors noted, suggests that buyout participation may be as much about neighborhood attainment—moving to places with greater social status or amenities—as retreating from environmental risk (ibid., p. 57). Residents from predominantly white neighborhoods tended to relocate within the same area or to other similarly white areas even when these were also sites of flood risk, while residents of more racially diverse neighborhoods tended to relocate to whiter destinations, revealing the racialized character of buyout-afforded mobility (ibid., p. 67).

Externally defined, “objective” measures offer only a partial picture of changes in living conditions; it is essential to also consider “subjective” measures of vulnerability, risk, and resilience that take into account people’s own perceptions, preferences, and self-assessments (Jones 2019). After Hurricane Katrina, for instance, some displaced New Orleans residents decided to return to the city despite their post-storm destinations seeming to offer a marked improvement in neighborhood quality and socioeconomic gains. As Asad (2015, p. 283) found, in such post-disaster decisions, “subjective experiences matter above and beyond these objective, place-specific considerations.” In other words, “a context’s objective causal efficacy—that is, whether it is good or bad according to objective measures such as having a high income and low levels of violence—was a secondary consideration” compared to “how individuals interacted with each context,” with experiences of discrimination in social, institutional, and labor market contexts forming one key factor in return decisions (ibid., p. 289).

Compared to the long-distance displacement experienced by many post-Katrina, participants in buyout programs tend to move very short distances (Loughran and Elliott 2019; McGhee et al. 2020). This may do less to reduce hazard exposure, but lower risks from social disruption, helping those who retreat to sustain local ties and support networks by remaining close to workplaces, schools, family, and friends. In the case of Staten Island, for example, it is possible that most buyout participants favored relocating nearby despite proximate areas ranking higher on various measures of social vulnerability. It is also worth considering whether participants moving from higher-income, whiter neighborhoods were contributing to neighborhood change, even displacement, in their destinations—a buyout-fueled form of “climate gentrification” (Keenan et al. 2018)—given that historically disinvested higher-ground parts of Staten Island were undergoing redevelopment after Sandy. At the same time, interviews with some participants reveal their difficulty accessing comparable housing elsewhere in the borough (Baker et al. 2018). Compensation exceeding pre-storm value may still not account for the lower cost of flood-prone housing, and the timing of an acquired property’s closing and “duplication of benefits” rules, which subtract prior disaster aid and insurance payments from the purchase price, are program elements that present additional barriers (ibid.).

The ability to realize post-disaster preferences, whether they be to remain, return, or relocate to a particular place, depends partly on material constraints and affordances, such as the level of support a recovery program provides. In managed retreat, this includes compensation, timing, and any assistance finding new housing, elements that not only vary from one program to another but also for different participants within the same program and post-disaster context. For example, an analysis of acquisitions following the 2008 Iowa floods found “that households in high social vulnerability areas,” especially those with high elderly and Hispanic populations, “were less likely to obtain full financial compensation, and endured longer periods before receiving acquisition funds,” even as some other households saw “extreme high recovery rates,” receiving more than pre-storm value (Muñoz and Tate 2016, p. 13). The appraisal and appeals process is just one element made easier to navigate and more likely to produce better outcomes, for “individuals with social connections and the political and economic power to access higher proportions of federal funds” (ibid.). Given the long history and ongoing practice of devaluing homes in communities of color and appraising comparable homes in white areas at higher values (Howell and Korver-Glenn 2020), unequal buyout outcomes rest on inequities that extend beyond any one disaster or recovery program. However, program design does little to acknowledge or redress this, typifying “colorblind adaptation” that deepens racialized divides and undermines the ability of marginalized groups to cope with environmental change (Hardy et al. 2017; Marino 2018).

Even in the face of repeat disasters, staying put persists as the default position in disaster and climate adaptation policy. Yet what it means to stay in place in the context of climate change is as varied, uncertain, and shifting as are the experiences and outcomes associated with planned relocation, warranting fresh attention from scholars. Studies underscore the psychological toll that a radically altered post-disaster landscape can take on residents who return or remain, particularly those who have lived in the location for a long time and possess stronger place attachment and local social ties (Haney and Gray-Scholz 2020). Meanwhile, terms such as “solastalgia” and “stationary displacement” or “displacement in place” speak to the psychically—and often physically—damaging consequences of witnessing one’s home and the surrounding environment degraded in ways that may be gradual but no less violent than sudden disruption (Albrecht et al. 2007; Nixon 2011). Concern for “trapped populations,” people who might prefer to move but confront obstacles to doing so (Black and Collyer 2014), joins calls to recognize and plan for the “voluntary immobility” of those who resist relocation as a threat to sovereignty, livelihoods, or cultural survival (Farbotko et al. 2020). Scholars in the emerging climate (im)mobilities field stress that moving and staying put should be understood as interrelated and not mutually exclusive; policies may facilitate both at once, and examining people’s experiences often reveals a complex blend of movement and stasis (Zickgraf 2019).

In the USA post-disaster, decisions to remain, like those to retreat, are subject to different, unevenly distributed levels of agency and government support that translate into a range of possible trajectories. One might seek to stay because no more appealing option is available or due to a sense of place attachment, identity, or dependence that makes alternatives less viable (Chamlee-Wright and Storr 2009). Rebuilding in place, especially for marginalized groups that have endured repeated attempts at dispossession, can be a political act—a sign and source of empowerment as much as vulnerability (Phillips et al. 2012; Martin 2019). But it also entails continued exposure for people and places not afforded sufficient resources, public investment, and infrastructure to protect from hazards, let alone climate change’s amplified impacts. As more extreme events strain existing systems of disaster recovery, and beget a range of place-based and other responses under the banner of adaptation, it is imperative to understand experiences of different recovery and adaptation pathways in diverse contexts.

## Research design and methods

### Sampling and study design

Survey design and data collection for this study were conducted on behalf of New York University by the Eagleton Center for Public Interest Polling (ECPIP). The *Life on the Coast: Before and After Hurricane Sandy Study* is a cross-sectional survey that examined items related to respondents’ living situations before and after Sandy (e.g., questions on time spent living in the pre-Sandy neighborhood; household composition and finances; perceptions and experiences of pre- and post-Sandy neighborhood social ties, social support, and flooding; expectations of future tenure); exposure to the storm and immediate impacts (e.g., house damage; length of displacement; number of moves; any income losses resulting from the storm); issues of rebuilding, relocation, and recovery (e.g., assistance received and debt accrued; perceptions of social, financial, and health circumstances relative to pre-Sandy period; feelings about different levels of government and participation in post-Sandy New York State, New York City, and/or New Jersey housing recovery programs; relocation versus rebuilding preferences and contributing factors; perceptions of neighborhood and community recovery); plus perceptions of climate change and mitigation and adaptation policies; and demographic information. Survey questions and sampled neighborhoods were informed by the lead author’s ethnographic fieldwork on Staten Island’s buyout process. The study sought to complement and extend fieldwork findings by generating data on comparable neighborhoods beyond Staten Island and following self-reported outcomes for a subset of homeowners.

To conduct a targeted neighborhood comparison of areas eligible and ineligible for buyout programs, including the NY Rising Buyout Program and New Jersey Blue Acres Program, the survey targeted 2001 parcel lots in 11 Sandy-affected parts of New York and New Jersey. Neighborhoods were strategically chosen to gather responses from homeowners potentially eligible for buyouts who expressed varying collective preferences for retreat versus rebuilding and had different recovery options. Alongside neighborhood-level comparison, we explored individual-level differences among those who rebuilt versus relocated, whether through a buyout or not.

Surveyed buyout areas included Oakwood Beach, where nearly two hundred households petitioned for buyouts after Sandy, which became the pilot site for the NY Rising Buyout Program in February 2013. This program offered homeowners in designated areas pre-storm value for their houses plus incentives for relocating nearby and participating as part of a targeted cluster of homes. Other buyout and wanted-buyout areas surveyed included neighborhoods where households similarly organized and lobbied for inclusion in the program. Portions of two of these neighborhoods plus an expanded section of Oakwood Beach were eventually deemed eligible. Others were not. This is a common but understudied experience, as demand for buyouts regularly outpaces available funding and political will to curtail or remove development.

For New York City residents inside and outside buyout areas, the city’s Build It Back program, launched in June 2013, offered rebuilding and elevation assistance as well as an alternative to buyouts for applicants who wished to relocate: the Acquisition for Redevelopment Program (NYSAfR), which purchased properties and auctioned them off to be redeveloped under stringent requirements rather than returned to natural open space as per the NY Rising Buyout Program. Beyond Staten Island, our rebuild-in-place areas include hard-hit parts of South Brooklyn and Queens where residents did not engage in the organized pursuit of buyouts, instead opting predominantly to apply for rebuilding assistance through Build It Back.

The panel of participants only included homeowners who resided in the predefined sample area at the time of Hurricane Sandy on October 29, 2012. By the time of the survey, 313 of the parcels were owned by the New York State Housing Trust Fund Corporation, which purchased properties for the buyout program. People no longer lived in these parcels, and efforts were made to reach homeowners who permanently relocated or temporarily moved by contacting secondary and forwarding addresses, phone numbers, and emails. Duplicate property owners were removed from the panel prior to fielding, resulting in 1876 property records.

ECPIP fielded the survey in mixed mode, including mail, online, and telephone with live callers, in part due to the challenge of locating a disaster-affected population, many of whom had relocated. Mail surveys were sent to panel subjects during February 2016 with the majority of responses received by March 2016. ECPIP used public records to augment the address-based panel with landline phone numbers and email addresses where available. The survey team contacted one-third of panel subjects for whom valid email addresses were available (*n* = 612), directing them to a web survey. On the final contact, respondents were entered into a drawing for one of five $50 gift cards if they completed the survey within 1 week. Telephone recruitment ran from March to mid-April 2016, with up to nine attempts to contact panel subjects with available phone numbers.

Completion rates varied by mode, with mail having a slightly higher rate (7%) than phone (6%) or online (4%). It is important to note that these completion rates are not comparable to industry standards since panelists surveyed were derived from records 3 years old, with only property owners who lived in the target areas at or before Hurricane Sandy included. The final numbers of responses by mode were 124 by mail, 63 by phone, and 38 by web, for a total of 225. Bias estimates were completed to ensure there were no statistically significant differences among respondents based on mode.

### Measures

#### Outcome measures

At the individual level, wellness serves as an indicator of how people or communities have recovered from a disruption and adapted to an altered environment (Norris et al. 2008). This is an important measure for policymakers concerned with protecting physical and behavioral health after a disaster (ibid.). The survey asked about the change in wellness as follows: “Please tell us if the following situations are *NOW* better, worse, or about the same as compared to *BEFORE* Sandy?” Situations included “physical health,” “mental/emotional health,” and “general stress level.” These analytical models use the latter, “general stress level,” because stress (e.g., psychological distress) is often used as a proxy of disaster recovery. The responses were dichotomized as “about the same” or “better” (0) and “worse” or no response (1) due to frequency distributions.

#### Main predictor variables

We examined the general stress level of residents using two main predictor variables: individual relocation decision and neighborhood category. Individual relocation decision was dichotomized between those who relocated compared to those who did not relocate (reference), regardless of neighborhood category. The second main predictor variable, neighborhood category, is a dummy variable indicating whether the individual lived in a buyout area, wanted-buyout area, or rebuild-in-place area (reference). Since individuals could make decisions that differed from the collective sentiment expressed in their area (as reflected in neighborhood category), we deemed it important to measure the effects of these two predictor variables.

Additionally, we considered variables relevant to self-reported stress and disaster recovery: house type and damage; whether the respondent took on debt due to Sandy; support from nearby friends and/or relatives; length of time in the neighborhood; flood insurance status; and current housing tenure. Damage was dichotomized as “undamaged” or “damaged but generally livable” (ref.) versus “damaged and unlivable for a month or more,” “destroyed completely,” or “condemned.” We considered the effect of taking on debt with a dichotomous variable comparing those who did and did not (reference). In setting up the survey, parcel type was recorded to provide information about whether the respondent lived in a single-family home or a different house type (reference). To account for social support, respondents were asked if they agreed or disagreed with the statement, “I have friends/relatives nearby I can rely on for help or support.” These were coded as agree and disagree (reference). Since duration is one proxy of place attachment (Merdjanoff 2013), respondents were asked how long they had lived in their neighborhood before Sandy. This continuous variable was dichotomized at the mean of 15 years. Those who had lived in their neighborhood for more than 15 years were coded as the reference group compared to those who had lived there for less. Because flood insurance could facilitate rebuilding and recovery, respondents were dichotomized into two groups: those who had flood insurance and those who did not (reference). To capture housing stability and recovery, respondents were asked whether they owned or rented at their current location. Those who owned were coded as 1 compared to all other responses (reference). Lastly, respondents were asked whether they had thought about climate change more often or less often/about the same (reference) since Sandy, as perceptions of climate change risk might influence whether a respondent relocated after Sandy and, if not, their future expectations of their neighborhood.

#### Sociodemographic controls

Sociodemographic control variables included demographic measures such as age (25–50 (ref.); 51–65; and 66+), gender (male (ref.); female), race (non-Hispanic White (ref.); African American; Hispanic/Latino; Asian/Pacific Islander; other; no response), household income before taxes (less than $50,000; greater than $50,000 (ref.)), current relationship status (partnered (ref.); single/separated/divorced/widowed/no response), and the number of children in the household at the time of the survey (at least one child (ref.); no children).

#### Analytic Plan

We analyzed bivariate associations to examine individual- and neighborhood-level differences. Adjusted logistic regressions were applied to test the association of predictors with individual relocation status and neighborhood category. The models included controls for demographic, socioeconomic, and political views. All analyses set significance levels at the *p* < =0.05 level. We used Stata statistical software, version 15. The Institutional Review Board of New York University approved the research.

## Results

### Bivariate analyses

Table 1 provides the bivariate descriptive statistics for predictor and sociodemographic control variables by individual-level relocation status and neighborhood category. Beginning with the individual status, a majority of respondents did not relocate after Sandy (74.6%) compared to those who did (25.4%). Overall, a majority of respondents reported that their general stress level had worsened since Sandy (54.0%).

**Table 1:** Descriptive Statistics at Individual and Neighborhood Level (column %; *n* = 225)

	All	Individual level		Neighborhood level		
		Did not relocate	Relocated	Buyout areas	Lobbied for buyout	Rebuild in place
Overall (column %)	100	74.6	25.4	26.8	25.3	47.9
General stress						
*Better/same since Sandy*	46.0	40.9**	61.1	54.4***	64.8	31.4
*Worse since Sandy*	54.0	59.1	38.9	45.6	35.2	68.6
Housing damage						
*Damaged/destroyed/condemned*	71.4	33.3**	14.8	82.5*	57.4	72.5
*Other*	28.6	66.7	85.2	17.5	42.6	27.5
Debt as result of Sandy						
*Yes*	60.6	62.3**	55.6	52.6**	46.3	72.5
*Other*	39.4	37.7	44.4	47.4	53.7	27.5
House type						
*One family residence*	60.1	60.4	59.3	47.4**	51.9	71.6
*Other*	39.9	39.6	40.7	52.6	48.1	28.4
Support from nearby friends/relatives						
*Yes*	79.3	79.3	79.6	75.4	77.8	82.3
*No*	20.7	20.7	20.4	24.6	22.2	17.7
Length of time in neighborhood						
*15 years or less*	51.2	50.9	51.8	49.1***	75.9	39.2
*More than 15 years*	48.8	49.1	48.2	50.9	24.1	60.8
Flood insurance						
*Yes*	62.4	74.8***	25.9	42.1***	63.0	73.5
*No*	37.6	25.2	74.1	57.9	37.0	26.5
Current homeowner						
*Yes*	89.2	94.3***	74.1	86.0	92.6	89.2
*No*	10.8	5.7	25.9	14.0	7.4	10.8
Think about climate change						
*More often since Sandy*	55.9	56.0	55.6	59.7	63.0	50.0
*Less often/about the same since Sandy*	44.1	44.0	44.4	40.3	37.0	50.0
Political Party Affiliation						
*Republican*	37.1	39.0	31.5	38.6	35.2	37.3
*Democrat*	21.1	18.9	27.8	24.6	13.0	23.5
*Independent*	16.4	17.0	14.8	19.3	16.7	14.7
*Undeclared/no answer*	25.4	25.2	25.9	17.5	35.2	24.5
Age						
*50 years and younger*	31.5	30.2	35.2	33.3	31.5	30.4
*51–65 years old*	40.8	42.8	35.2	38.6	37.0	44.1
*66 years and older*	27.7	27.0	29.6	28.1	31.5	25.5
Household income						
*Greater than 50K*	71.8	28.3	27.8	73.7	64.8	74.5
*Other*	28.2	71.7	72.2	26.3	35.2	25.5
Race/ethnicity						
*White*	82.2	80.5	87.0	86.0	85.2	78.4
*Non-White*	17.8	19.5	13.0	14.0	14.8	21.6
Marital status						
*Married/partnered*	67.6	67.3	68.5	63.2*	57.4	75.5
*Separated/divorced/widowed/single*	32.4	32.7	31.5	36.8	42.6	24.5
Gender						
*Male*	51.2	50.3	53.7	54.4*	35.2	57.8
*Female*	48.8	49.7	46.3	45.6	64.8	42.2
Children in the household						
*Yes*	46.0	42.1	57.4	57.9	44.4	40.2
*No*	54.0	57.9	42.6	42.1	55.6	59.8

*p < 0.05

**p < 0.01

***p < 0.001

There were important differences between those who relocated and those who did not, although no significant sociodemographic differences were observed. A significantly higher percentage of respondents who did not relocate reported that their stress had worsened (59.1%) compared to about 39% of those who did relocate. A higher percentage of those with damaged, destroyed, or condemned homes did not relocate (33.3%) compared to those who did relocate (14.8%). Those who did not relocate were more likely to take on debt as a result of Sandy (62.3%) while about 56% of those who relocated took on debt. Overall, nearly 60% of respondents took on debt. Approximately 75% of those who did not relocate had flood insurance compared to only 26% of those did relocate. Nearly all respondents who did not relocate indicated that they were current homeowners (94%), compared to 74% of those who relocated.

At the neighborhood level, nearly half of the respondents lived in rebuild-in-place areas (47.9%). About 26.8% were from buyout areas and 25% from wanted-buyout areas. Those living in neighborhoods that rebuilt in place with no organized buyout effort indicated the highest percent of worsened stress among all categories (68.6%). This was a statistically significant difference as about 46% of those from buyout areas and 35% of those from wanted-buyout areas reported that their stress had worsened since Sandy.

There were also statistically significant neighborhood-level differences when it came to housing damage and debt. Respondents from rebuild-in-place areas reported being highly impacted by the storm, with nearly 72% reporting that their homes were either damaged so badly they were unlivable, destroyed, or condemned, and approximately 73% reporting taking on debt as a result of Sandy. About 83% of respondents from buyout areas reported similarly high levels of damage, but only a little over half (52.6%) reported taking on debt. About 57% of respondents from wanted-buyout areas sustained similarly high damage, which suggests that damage may have been tied to whether or not neighborhoods were bought out. About 46% of respondents from wanted-buyout areas reported taking on debt due to the storm.

Another statistically significant difference concerned house type. Nearly 72% of respondents from rebuild-in-place areas reported living in a one-family residence at the time of Sandy, versus 47.4% and 51.9% in buyout and wanted-buyout areas, respectively. Slightly more than 60% of those from rebuild-in-place areas reported that they had lived in their neighborhoods for more than 15 years, compared to 51% of those from buyout areas and 23% of those in wanted-buyout areas. As with individual relocation status, significant differences existed among neighborhood types when it came to holding flood insurance at the time of Sandy: overall, about 60% of respondents had flood insurance, but this rose to 73% of those from rebuild-in-place areas and dropped to about 42% for those from buyout areas.

Finally, there were significant group differences for two sociodemographic control variables: marital status and gender. A higher percentage of those from rebuild-in-place areas were married or partnered (75.5%), compared to 63.2% of those from buyout areas and 57.4 of those from wanted-buyout areas. A majority of male respondents lived in buyout areas (54.4%) or rebuild-in-place areas (57.8%), with 35.2% from wanted-buyout areas.

### Logistic regressions

Table 2 presents the odds ratios from multivariate logistic regression examining the association between self-reported stress, the two main independent variables, and additional covariates. Model 1 examines the association between stress and individual relocation status. The adjusted logistic regression results demonstrate that the individual decision to relocate after Sandy significantly decreased the likelihood of reporting worsened stress. Those who relocated were 75% less likely to report worsened stress than those who stayed in place.

**Table 2:** Multivariate logistic regressions for the association of stress with individual relocation status and neighborhood category, and covariates

	Adjusted odds ratio for individual relocation decision OR (CI)	Adjusted odds ratio for neighborhood category OR (CI)
*Independent variables*		
Individual decision		
*Relocated*	0.25 (0.10,0.64)**	–
*Stayed in place*	*ref.*	–
Neighborhood buyout status		
*Category 1 (eligible for buyout)*	–	0.36 (0.15, 0.87)*
*Category 2 (lobbied buyout)*	–	0.24 (0.09, 0.61)**
*Category 3 (rebuild in place)*	–	*ref.*
Housing damage		
*Damaged/destroyed/condemned*	3.32 (1.53, 7.20)**	2.73 (1.26, 5.89)*
*Other*	*ref.*	*ref.*
Debt as a result of Sandy		
*Yes*	3.02 (1.47, 6.19)***	2.23 (1.09, 4.57)*
*Other*	*ref.*	*ref.*
House type		
*One family residence*	2.20 (1.12, 4.30)*	1.50 (0.74, 3.03)
*Other*	*ref.*	*ref.*
Support from nearby friends/relatives		
*Yes*	0.16 (0.06, 0.41)***	0.17 (0.07, 0.44)***
*No*	*ref.*	*ref.*
Length of time in neighborhood		
*15 years or less*	1.93 (0.95, 3.92)	1.49 (0.71, 3.11)
*More than 15 years*	*ref.*	*ref.*
Flood insurance		
*Yes*	0.95 (0.41, 2.21)	1.49 (0.69, 3.24)
*No*	*ref.*	*ref.*
Current homeowner		
*Yes*	0.29 (0.09, 0.96)*	0.50 (0.16, 1.55)
*No*	*ref.*	*ref.*
Think about climate change		
*More often since Sandy*	1.71 (0.86, 3.39)	1.82 (0.91, 3.65)
*Less often/about the same since Sandy*	*ref.*	*ref.*
Political Party Affiliation		
*Republican*	0.82 (0.33, 2.00)	0.97 (0.39, 2.40)
*Democrat*	0.60 (0.22, 1.63)	0.57 (0.21, 1.57)
*Independent*	0.59 (0.20, 1.74)	0.75 (0.26, 2.19)
*Undeclared/no answer*	*ref.*	*ref.*
Age		
*50 years and younger*	*ref.*	*ref.*
*51–65 years old*	1.44 (0.63, 3.30)	1.46 (0.62, 3.42)
*66 years and older*	0.49 (0.18, 1.35)	0.55 (0.20, 1.51)
Household income		
*Greater than 50K*	0.61 (0.26, 1.40)	0.54 (0.23, 1.27)
*Other*	*ref.*	*ref.*
Race/ethnicity		
*White*	0.93 (0.40, 2.16)	1.07 (0.45, 2.51)
*Non-White*	*ref.*	*ref.*
Marital status		
*Married/partnered*	0.93 (0.41, 2.12)	0.64 (0.27, 1.49)
*Separated/divorced/widowed/single*	*ref.*	*ref.*
Gender		
*Male*	1.35 (0.68, 2.71)	1.05 (0.52, 2.13)
*Female*	*ref.*	*ref.*
Children in the household		
*Yes*	0.97 (0.46, 2.04)	1.04 (0.49, 2.23)
*No*	*ref.*	*ref.*
Total *N*	213	213
Pseudo *R*^2^	0.2241	0.2297

*p < 0.05

**p < 0.01

***p < 0.001

Those whose homes were severely damaged, destroyed, or condemned were approximately 3.3 times more likely to report higher levels of stress since Sandy. Respondents who reported that they took on debt as a result of the storm were three times more likely to report worsened stress than those who did not take on debt. Living in a single-family residence was also tied to stress, as these respondents were more than two times as likely to report worsened stress as those not living in a single-family residence.

Having support from nearby friends and/or relatives was protective, with those who indicated having such social support about 85% less likely to report worsened stress. Being a current homeowner was also protective. Current homeowners were about 70% less likely to report worsened stress than those who did not own their homes. No sociodemographic controls were significantly associated with worsened stress; however, the fully adjusted model accounts for a reasonable amount of variance (*R*^2^ = 0.22).

Model 2 is the fully adjusted logistic regression examining the relationship between general stress, neighborhood-level buyout status, and covariates. Those who lived in buyout-eligible neighborhoods were 66% less likely to report worsened stress compared to those from rebuild-in-place neighborhoods. Those who lived in wanted-buyout neighborhoods were also significantly less likely (OR = 0.24) than those from rebuild-in-place neighborhoods to report worsened stress.

Many of the significant associations found in model 1 are also present in model 2. For instance, those whose homes were severely damaged, destroyed, or condemned were 2.7 times more likely to report worsened stress. Debt was again a strong predictor of stress. Respondents who reported taking on debt as a result of Sandy were about 2.2 times more likely to report worsened stress since than storm than were those who did not take on debt. Lastly, support from nearby friends and/or relatives remained protective. Those with this type of support were 83% less likely to experience worsened stress than were those who lacked such support. Overall, the model accounts for a similar amount of variance as model 1 (*R*^2^ = 0.23).

## Study limitations

The post-Sandy buyout and rebuilding processes were slow-moving and often unclear. Alongside the patchiness characteristic of disaster data more generally (Liboiron 2015), this made it challenging to create a sampling frame, locate respondents, and compile survey data, contributing to a low response rate. Although our data are not representative of all homeowners living in affected areas at the time of Sandy, they do allow for a targeted comparison of areas eligible and ineligible for government-funded buyouts. The low response rate is a limitation and led to the dichotomization of certain variables. Still, we believe our results are an important step toward understanding the mid- to long-term effects of different housing recovery pathways and make a contribution to existing research on property buyouts that is similarly hampered by patchy data at the federal and program levels (Mach et al. 2019).

## Discussion and conclusions

This study’s goal was to explore the outcomes of post-disaster relocation actions and program options at individual and neighborhood levels. We analyzed data from survey respondents from Sandy-affected neighborhoods that either received a government-funded buyout option based on local demand; organized and lobbied for a buyout option unsuccessfully; or saw no organized buyout effort. The results indicate a relationship between relocation and reduced stress, with individuals who relocated or who lived in areas eligible for a buyout significantly less likely to report worsened stress. This is surprising given the extensive prior research showing relocation to be associated with adverse mental health outcomes. However, as noted above, nearly all prior research examines forced relocation, including studies of those displaced with little to no financial support or, if compensated in some way, with little opportunity to exercise agency over the relocation decision, process, or destination.

The situation examined here differed. Although the storm represented a loss of control, the subsequent experience of successfully seeking a buyout or opting to relocate independently may have contributed to restoring it. New York State’s buyout program was more financially generous than many such programs, due partly to the high cost of housing in the New York metropolitan area. And while state support was primarily financial in nature, lacking the explicit “logic of care” of sustained, holistic engagement (De Vries and Fraser 2017), residents eligible for buyouts had the promise of significant compensation and opportunity to escape not just physical risk and exposure but also emotional triggers associated with the disaster site.

Previous research has shown that housing damage is linked to mental health, but it is not a simple correlation (Ursano et al. 2008; Merdjanoff 2013). For example, Merdjanoff found that Hurricane Katrina survivors whose homes were completely destroyed reported *less* emotional distress than those whose homes were damaged so much that they were uninhabitable, perhaps because deciding what to do with a damaged house and possessions is even more draining than being forced to move forward with finding a new home and starting over (2013). This theory may apply to relocation. Experiencing and re-experiencing the damage of one’s home and neighborhood on an everyday basis can represent continuous emotional trauma. Residents who were able to relocate to undamaged homes and areas where they felt safer could avoid the incessant reminders of Sandy that persisted through the time of our survey. In the lead author’s ethnographic fieldwork, for instance, she encountered one Staten Island buyout participant who agreed to an interview only at a location not within sight of the water or his former home. This suggests that those who stayed put, whether by choice or because they lacked the ability to sell or resources to move without financial support, were not afforded the opportunity for emotional closure a buyout might provide.

At the same time, other studies of relocated buyout participants—including participants in the same post-Sandy program—paint a different picture. Baker et al. (74) find that Staten Island participants’ sense of agency decreased over the course of the program, which introduced new stressors, including final compensation amounts that proved insufficient for some residents to start over in a comparable location. Due to the lengthy timeline of most buyout programs, it is likely that some of our survey participants were still in the midst of leaving their old homes and transitioning to new ones; more longitudinal studies are needed to understand potential increases in stress over time for those who move, as they may struggle to develop ties in new places (Binder et al. 2019), or face increased housing instability in the future (Elliott and Howell 2017). While Oakwood Beach residents reported satisfaction with the buyout when surveyed in general terms, interviews revealed mixed feelings (Baker et al. 2018). Such studies show experiences of retreat to be complex and nuanced, involving trade-offs and lingering effects unlikely to be readily apparent even to participants themselves.

While it will require further research to ascertain the conditions under which relocation is associated with positive outcomes for those involved, the results from model 1 reveal that those who rebuilt in place remained far from recovered more than 3 years after the storm. Residents seeking to rebuild faced many obstacles that could have increased the likelihood of experiencing worsened stress. Problems plagued New York City’s Build It Back program. Close to 2 years after Sandy, just 686 applicants––roughly 3% of the total––had received any financial assistance from the program, which was by then on its third director (the first of whom labeled Build It Back a “categorical failure”) (Landa 2015; Rizzi 2016). The program’s flaws, delineated in an audit by the city’s comptroller, were egregious in this case but indicative of a broader trend in government disaster-recovery programs contracted out to for-profit companies with little regulation or oversight. As Adams found in her study of market-driven recovery after Hurricane Katrina, “homeowners were forced to go into debt, arbitration, and lawsuits to recover funds they were promised” (2013). Homeowners faced similar struggles after Hurricane Sandy.

Compounding the difficulty was the challenge of flood insurance. Even residents who had flood insurance prior to Sandy did not necessarily receive sufficient funds to rebuild without taking on debt. Investigative reporting in 2015 also revealed that some of the engineers hired by insurance companies to evaluate flood damage falsified their reports to lower payouts (Chen 2015; Alfonsi 2015). In response, FEMA, which administers the National Flood Insurance Program, agreed to reevaluate Sandy claims upon request––adding another step to an already cumbersome process. Such experiences lend disaster recovery a cyclical character, punctuated by events that “renew feelings of disruption and disorientation,” though they may give rise to enhanced social cohesion and associated positive emotions too, should residents come together in response (Silver and Grek-Martin 2015, p. 40).

It is possible that people who stayed put after Sandy were trading off greater short-term stress for the benefits of a stronger sense of community and attachment to place. Yet even as post-storm stressors linked to applying for aid and claiming insurance were likely to diminish over time, residents who rebuilt faced profound uncertainty about the longer-term viability of their homes and neighborhoods. Shortly before Sandy, Congress passed the Biggert-Waters Flood Insurance Reform Act of 2012, setting in motion rate increases that would have proved unaffordable for numerous homeowners in the sampled neighborhoods. The 2014 Homeowner Flood Insurance Affordability Act slowed these increases pending further study, but the looming decrease in public subsidies and support for those living in flood-prone areas remained a palpable concern in neighborhoods like those surveyed (Elliot 2017).

Another potential source of stress even for those able to rebuild and afford flood insurance was the anticipation of future climate change. Our results reveal that only around half of respondents reported thinking about climate change more since Sandy, but there was a substantial decline in the number who said they expected to live in their neighborhood for a long time. While rebuilding plays an important role in the process of “reorientation” following a disaster’s disruption (Cox and Perry 2011; Silver and Grek-Martin 2015), the renewed sense of belonging, security, and place attachment we might expect to result from reconstructing familiar landscapes in concert with one’s neighbors may have been undermined by the anticipation of future threats. Experiencing worsening flooding and witnessing disasters across the country straining the resources put forward to respond and adapt, residents who rebuilt in place faced a future of increased reliance on external assistance allocated on a competitive, ad hoc basis; protective measures whose costs they might bear or that are not guaranteed to materialize and be maintained; and familiar surroundings becoming irrevocably altered as the waters rise.

Interestingly, respondents from neighborhoods that lobbied for buyouts but did not receive them were less likely to report worsened stress than respondents from rebuild-in-place areas. This is an important finding because a majority of respondents who lived in wanted-buyout areas reported that they stayed in place (82%). Yet they also reported less housing damage and subsequent debt than respondents from buyout and rebuild-in-place areas, likely contributing to their lower rates of stress. They reported living in their neighborhoods for shorter periods of time as well, whereas respondents from rebuild-in-place areas reported the longest length of residence and the most worsened stress since the storm.

Though the length of residence is an imperfect proxy for place attachment, returning to a pre-disaster home can be especially fraught for those whose greater emotional attachment to place amplifies the disconnect between a remembered state and post-disaster reality (Morrice 2013). Ensuing pain can prompt decisions to relocate and increased stress for those who stay, who may go on to witness the dissolution of their social as well as environmental surroundings. What results are “hollow homes,” a term coined by Tschakert, Tutu, and Alcaro “to indicate both environmental degradation and the deterioration of social networks” (2013, p. 18). Among our respondents, perceptions of proximate social support translated into greatly reduced stress levels; hence, the actual or anticipated loss of ties for those who remain—or ultimately opt to relocate—is of significant concern (maybe most so for communities that reside adjacent to buyout areas but are not included in them [Martin 2019; Barile et al. 2020; Binder et al. 2020]).

Neighborhood-level differences we observed suggest that it is not just the individual-level rebuild or relocation decision that influences post-disaster stress but also community-level organizing. Residents who collectively lobbied for a buyout gathered for meetings and shared information with each other, which may have fostered a stronger sense of community within the neighborhood. Organizing for a buyout, regardless of the outcome, could also have helped develop bridging forms of social capital--links with outside groups and actors that can facilitate disaster recovery (Aldrich 2012). Whether residents united to seek buyouts, and their subsequent stress, could also reflect preexisting differences in social capital and the “social infrastructure” that supports its development (Klinenberg 2018), although we might expect areas with weaker ties to be more likely to see willingness to retreat.

The bottom-up push for neighborhood-wide buyouts observed after Sandy and reflected in our data was unusual. Buyouts are typically conceived and implemented as individual transactions, with program design more likely to undermine collective action than facilitate it (Marino 2018; Elliott et al. 2020). There is, however, a growing push for policies that enable managed retreat at the community scale (GAO 2020). Future research can follow the effects of such policies, examine experiences of the process of collective resettlement, and explore how “community” is defined and contested in practice (Jessee 2020).

Organizing to envision and bring about transformational adaptation in the form of retreat has the potential to prove empowering; it presents an opportunity to remove houses from harm’s way, buffer adjacent areas, and restore habitat, mitigating risk locally and leaving a lasting legacy, even if unable to undo a disaster’s damage or alone address drivers of vulnerability and climate change. Any associated sense of agency and capacity to act can lessen negative emotions such as solastalgia, which emerges not simply from experiencing environmental change but from a perceived lack of control over that change (Albrecht et al. 2007; Tschakert et al. 2013). Joining this may be feelings of injustice or betrayal, “a loss of trust in the social institutions that are supposed to protect and secure people and their place in society” (Askland and Bunn 2018, p. 21). Receiving government support for managed retreat, or to enable voluntary immobility, may entail leaving or transforming a physical place but sustaining one’s place in a more expansive sense—for instance, through buyouts, which provide value for a primary asset, one’s house, otherwise at risk, or via other policies that support movement as a means to stability, enabling people to remain nearby for longer, at lesser risk (Zickgraf 2019).

Buyouts present a form of security available primarily to those who already hold a degree of social, political, and economic power (Marino 2018; Koslov 2019). Respondents to our survey were homeowners, predominantly white with incomes higher than $50,000, and thus reflective of groups that generally fare far better after disasters than many others (but see Madajewicz 2020). The New York metropolitan region’s renters, disproportionately poor and people of color long excluded from the benefits of government-backed homeownership, likewise did not benefit from the option to participate in a buyout program. Rather than being able to decide whether to rebuild or move with such support, renters faced an especially great risk of forced relocation and long-term displacement. They were more likely to have difficulty accessing recovery assistance and to wind up paying higher rents for more crowded housing (Make The Road 2014). As is true of other disasters and climate change generally, stressors affecting our respondents were not distributed or encountered equally nor were resources to mitigate, adapt to, and cope with them.

The common struggles and emerging divides that characterize experiences of staying put or relocating after disasters, and in light of climate change, remain far from understood. Access to buyouts or other voluntary and supported managed retreat is only one piece of the puzzle, but one that will likely grow in importance. Despite little data as yet on the outcomes of buyout programs for affected individuals and communities, such programs are increasing in popularity and potential scale. After Hurricane Harvey in 2017, more than 3500 homeowners submitted applications to participate in a Harris County, Texas, buyout program (Patterson 2017, p. 3). In Louisiana, a Coastal Master Plan released the same year suggested buying out more than 2000 homes at the highest risk of flooding (Wendland 2018). Meanwhile, proposed reforms to the National Flood Insurance Program include ramping up federal funding for buyouts, particularly of recurrently flooded properties (Moore 2017). Yet, so far at least, buyout programs that have taken place are actually decreasing in size (Mach et al. 2019). Should more co-occurring and costly disasters strain resources—particularly in light of Covid-19—experiences of staying put without the option to move elsewhere may become even more prominent.

Climate change’s consequences for mental health and well-being make up a budding area of study, not least in relation to climate-linked relocation and migration (Schwerdtle et al. 2018; Berry et al. 2018; Dannenberg et al. 2019). Mediating these consequences will be access to managed retreat programs alongside factors identified here, including debt-based disaster assistance, increases in insurance rates, proximity of social support, and mechanisms of collective empowerment and self-determination that together shape the extent to which people and places affected by climate change transform in ways that are sustainable. Although this study has limitations, it is a step in building a line of inquiry regarding the longer-term experiences of those who participate in buyout programs, retreat in less “managed” ways, or stay put after extreme events projected to become more frequent and severe in coming years.

## Data Availability

The data are not publicly available because they contain information that could compromise research participant privacy/consent.

## Footnotes

1 Binder et al. (2019) conducted a similar post-Sandy study, surveying respondents from three New York City neighborhoods—one that received a buyout, one adjacent to the buyout zone, and one that prioritized rebuilding in place—and comparing measures of place attachment, social capital, and risk perception. Our study, conducted at approximately the same time, complements this by drawing from a larger sample of neighborhoods—multiple buyout areas, plus nearby, though not adjacent, areas where residents sought buyouts, and three areas focused on rebuilding with no buyout option—and comparing a range of social, economic, and health outcomes.

## References

[CR1] Adams V (2013). Markets of sorrow, labors of faith: New Orleans in the wake of Katrina.

[CR2] Albrecht G, Sartore G-M, Connor L et al (2007) Solastalgia: the distress caused by environmental change. Australasian Psych 15(sup1):S95–S98. 10.1080/10398560701701288 10.1080/10398560701701288 18027145

[CR3] Aldrich DP (2012). Social, not physical, infrastructure: the critical role of civil society after the 1923 Tokyo earthquake. Disasters.

[CR4] Alfonsi S (2015) The storm after the storm. 60 Minutes. CBS. WCBS, New York: Jun 7. Television

[CR5] AP-NORC Center for Public Affairs Research (2013) After a Disaster Strikes: Public Opinion on Rebuilding and Relocation Policies (Issue Brief). July. https://apnorc.org/wp-content/uploads/2020/02/Rebuild-or-Relocate_FINAL-FOR-POST_fxd.pdf

[CR6] Arcaya M, Raker EJ, Waters MC (2020). The social consequences of disasters: individual and community change. Annu Rev Sociol.

[CR7] Asad AL (2015). Contexts of reception, post-disaster migration, and socioeconomic mobility. Popul Environ.

[CR8] Askland HH, Bunn M (2018). Lived experiences of environmental change: solastalgia, power and place. Emot Space Soc.

[CR9] Baker CK, Binder SB, Greer A (2018). Integrating community concerns and recommendations into home buyout and relocation policy. Risk, Hazards & Crisis in Public Policy.

[CR10] Barile JP, Binder SB, Baker CK (2020). Recovering after a natural disaster: differences in quality of life across three communities after hurricane Sandy. Applied Research Quality Life.

[CR11] Berry HL, Waite TD, Dear KBG (2018). The case for systems thinking about climate change and mental health. Nat Clim Chang.

[CR12] Binder SB, Greer A (2016). The devil is in the details: linking home buyout policy, practice, and experience after Hurricane Sandy. Politics and Governance.

[CR13] Binder SB, Baker CK, Barile JP (2015). Rebuild or relocate? Resilience and postdisaster decision-making after Hurricane Sandy. Am J Community Psychol.

[CR14] Binder SB, Barile JP, Baker CK, Kulp B (2019). Home buyouts and household recovery: neighborhood differences three years after Hurricane Sandy. Environmental Hazards.

[CR15] Binder SB, Ritchie LA, Bender R (2020). Limbo: the unintended consequences of home buyout programmes on peripheral communities. Environmental Hazards.

[CR16] Black R, Collyer M (2014). Populations ‘trapped’ at times of crisis | forced migration review. FMR.

[CR17] Chamlee-Wright E, Storr VH (2009). “There’s no place like New Orleans”: sense of place and community recovery in the Ninth Ward after Hurricane Katrina. J Urban Aff.

[CR18] Chen DW (2015) Hurricane Sandy victims say damage reports were altered. The New York Times

[CR19] Cox RS, Perry K-ME (2011). Like a fish out of water: reconsidering disaster recovery and the role of place and social capital in community disaster resilience. Am J Community Psychol.

[CR20] Dannenberg AL, Frumkin H, Hess JJ, Ebi KL (2019). Managed retreat as a strategy for climate change adaptation in small communities: public health implications. Clim Chang.

[CR21] De Vries DH, Fraser JC (2012). Citizenship rights and voluntary decision making in post-disaster U.S. floodplain buyout mitigation programs. Int J Mass Emerg Disasters.

[CR22] De Vries DH, Fraser JC (2017). Historical waterscape trajectories that need care: the unwanted refurbished flood homes of Kinston’s devolved disaster mitigation program. J Polit Ecol.

[CR23] Elliot R (2017). Who pays for the next wave? The American welfare state and responsibility for flood risk. Polit Soc.

[CR24] Elliott JR, Howell J (2017). Beyond disasters: a longitudinal analysis of natural hazards’ unequal impacts on residential instability. Soc Forces.

[CR25] Elliott JR, Brown PL, Loughran K (2020). Racial inequities in the Federal buyout of flood-prone homes: a nationwide assessment of environmental adaptation. Socius.

[CR26] Farbotko C, Dun O, Thornton F (2020). Relocation planning must address voluntary immobility. Nat Clim Chang.

[CR27] Flavelle C (2020) U.S. flood strategy shifts to ‘unavoidable’ relocation of entire neighborhoods. New York Times. Aug 26.https://www.nytimes.com/2020/08/26/climate/flooding-relocation-managed-retreat.html

[CR28] GAO (2020) Climate change: A climate migration pilot program could enhance the nation’s resilience and reduce federal fiscal exposure. https://www.gao.gov/products/GAO-20-488

[CR29] Haney TJ, Gray-Scholz D (2020). Flooding and the “new normal”: what is the role of gender in experiences of post-disaster ontological security?. Disasters.

[CR30] Hardy RD, Milligan RA, Heynen N (2017). Racial coastal formation: the environmental injustice of colorblind adaptation planning for sea-level rise. Geoforum.

[CR31] Hino M, Field CB, Mach KJ (2017). Managed retreat as a response to natural hazard risk. Nat Clim Chang.

[CR32] Horton R, Little C, Gornitz V (2015). New York City panel on climate change 2015 report chapter 2: sea level rise and coastal storms: NPCC 2015 report chapter 2. Ann N Y Acad Sci.

[CR33] Howell J, Korver-Glenn E (2020) The increasing effect of neighborhood racial composition on housing values, 1980–2015. Soc Probl. 10.1093/socpro/spaa033

[CR34] Jessee N, Laska S (2020). Community resettlement in Louisiana: learning from histories of horror and Hope. Louisiana’s response to extreme weather: a coastal state’s adaptation challenges and successes.

[CR35] Jones L (2019). Resilience isn’t the same for all: comparing subjective and objective approaches to resilience measurement. WIREs Climate Change.

[CR36] Keenan JM, Hill T, Gumber A (2018). Climate gentrification: from theory to empiricism in Miami-Dade County, Florida. Environ Res Lett.

[CR37] Klinenberg E (2018). Palaces for the people: how social infrastructure can help fight inequality, polarization, and the decline of civic life.

[CR38] Klinenberg E, Araos M, Koslov L (2020). Sociology and the climate crisis. Annu Rev Sociol.

[CR39] Koslov L (2016) The case for retreat. Publ Cult 28(2):359–387. 10.1215/08992363-3427487

[CR40] Koslov L (2019) Avoiding climate change: Agnostic adaptation and the politics of public silence. Annals of the Amer Assoc of Geog 109(2):568-580. 10.1080/24694452.2018.154947

[CR41] Landa M (2015) Audit report on the administration of the New York City Build It Back Single Family Program by the Mayor’s Office of Housing Recovery Operations. City of New York Office of the Comptroller

[CR42] Liboiron M (2015). Disaster data, data activism : grassroots responses to representing Superstorm Sandy. Extreme weather and global media.

[CR43] Loughran K, Elliott JR (2019). Residential buyouts as environmental mobility: examining where homeowners move to illuminate social inequities in climate adaptation. Popul Environ.

[CR44] Mach KJ, Kraan CM, Hino M (2019). Managed retreat through voluntary buyouts of flood-prone properties. Sci Adv.

[CR45] Madajewicz M (2020). Who is vulnerable and who is resilient to coastal flooding? Lessons from Hurricane Sandy in New York City. Clim Chang.

[CR46] Make The Road (2014) Treading water: renters in post-Sandy New York. Make the Road

[CR47] Marino E (2018). Adaptation privilege and voluntary buyouts: perspectives on ethnocentrism in sea level rise relocation and retreat policies in the US. Glob Environ Chang.

[CR48] Martin A (2019) Race, place, and resilience: Social equity in North Carolina’s post-disaster buyout program [Unpublished PhD dissertation]. The University of North Carolina at Chapel Hill

[CR49] McGhee DJ, Binder SB, Albright EA (2020). First, do no harm: evaluating the vulnerability reduction of post-disaster home buyout programs. Natural Hazards Review.

[CR50] Merdjanoff AA (2013). There’s no place like home: examining the emotional consequences of hurricane Katrina on the displaced residents of New Orleans. Soc Sci Res.

[CR51] Moore R (2017) Seeking higher ground: how to break the cycle of repeated flooding with climate-smart flood insurance reforms. Natural Resources Defense Council

[CR52] Morrice S (2013). Heartache and Hurricane Katrina: recognising the influence of emotion in post-disaster return decisions. Area.

[CR53] Muñoz CE, Tate E (2016) Unequal recovery? Federal Resource Distribution after a Midwest Flood Disaster. Int J Environ Res Public Health 13. 10.3390/ijerph13050507 10.3390/ijerph13050507 PMC4881132 27196921

[CR54] Nixon R (2011). Slow violence and the environmentalism of the poor.

[CR55] NJ Department of Community Affairs (2013) Community development block grant disaster recovery action plan. https://www.renewjerseystronger.org/plans-reports/#cdbg

[CR56] Norris, FH, Stevens SP, Pfefferbaum B, et al (2008) Community resilience as a metaphor, theory, set of capacities, and strategy for disaster readiness. Am J Community Psychol 41(1–2):127–50. 10.1007/s10464-007-9156-6 10.1007/s10464-007-9156-6 18157631

[CR57] NY State Homes and Community Renewal (2013) State of New York action plan for community development block grant program disaster recovery. https://stormrecovery.ny.gov/sites/default/files/documents/CDBGActionPlan.pdf

[CR58] Pais JF, Elliott JR (2008). Places as recovery machines: vulnerability and neighborhood change after major hurricanes. Social Forces.

[CR59] Patterson G (2017) Case studies in floodplain buyouts: Looking to best practices to drive the conversation in the Houston region. Rice University Kinder Institute for Urban Research. https://kinder.rice.edu/sites/g/files/bxs1676/f/documents/KI%202018%20Buyout%20Report%20.pdf

[CR60] Phillips B, Stukes PA, Jenkins P (2012). Freedom Hill is not for sale—and neither is the lower Ninth Ward. J Black Stud.

[CR61] Riad JK, Norris FH (1996). The influence of relocation on the environmental, social, and psychological stress experienced by disaster victims. Environ Behav.

[CR62] Rizzi N (2016) Build it Back was a “categorical failure,” its creator says. DNAinfo

[CR63] Robinson CS, Davidson RA, Trainor JE (2018). Homeowner acceptance of voluntary property acquisition offers. International Journal of Disaster Risk Reduction.

[CR64] Rumbach A, Sullivan E, Makarewicz C (2020). Mobile home parks and disasters: understanding risk to the third housing type in the United States. Nat Hazards Rev.

[CR65] Schwerdtle P, Bowen K, McMichael C (2018). The health impacts of climate-related migration. BMC Med.

[CR66] Seebauer S, Winkler C (2020). Should I stay or should I go? Factors in household decisions for or against relocation from a flood risk area. Glob Environ Chang.

[CR67] Siders AR, Hino M, Mach KJ (2019). The case for strategic and managed climate retreat. Science.

[CR68] Silver A, Grek-Martin J (2015). “Now we understand what community really means”: reconceptualizing the role of sense of place in the disaster recovery process. J Environ Psychol.

[CR69] Solecki W, Leichenko R, Eisenhauer D (2017). Extreme climate events, household decision-making and transitions in the immediate aftermath of Hurricane Sandy. Miscellanea Geographica.

[CR70] Sullivan KD, Uccellini LW (2013) Service assessment: Hurricane/post-tropical cyclone Sandy, October 22–29, 2012. U.S. Department of Commerce, NOAA, and NWS, Silver Spring, Maryland. http://www.nws.noaa.gov/os/assessments/pdfs/Sandy13.pdf

[CR71] Tschakert P, Tutu R, Alcaro A (2013). Embodied experiences of environmental and climatic changes in landscapes of everyday life in Ghana. Emot Space Soc.

[CR72] Ursano RJ, Fullerton CS, Terhakopian A (2008). Disasters and health: distress, disorders, and disaster behaviors in communities, neighborhoods, and nations. Soc Res.

[CR73] Uscher-Pines L (2009). Health effects of relocation following disaster: a systematic review of the literature. Disasters.

[CR74] Wendland T (2018) Louisiana says thousands should move from vulnerable coast, but can’t pay them. All Things Considered

[CR75] Zickgraf C (2019). Keeping people in place: political factors of (im)mobility and climate change. Soc Sci.

